# Improved Somatic Mutagenesis in Zebrafish Using Transcription Activator-Like Effector Nucleases (TALENs)

**DOI:** 10.1371/journal.pone.0037877

**Published:** 2012-05-24

**Authors:** Finola E. Moore, Deepak Reyon, Jeffry D. Sander, Sarah A. Martinez, Jessica S. Blackburn, Cyd Khayter, Cherie L. Ramirez, J. Keith Joung, David M. Langenau

**Affiliations:** 1 Molecular Pathology Unit and Center for Cancer Research, Massachusetts General Hospital, Charlestown, Massachusetts, United States of America; 2 Harvard Stem Cell Institute, Boston, Massachusetts, United States of America; 3 Department of Pathology, Harvard Medical School, Boston, Massachusetts, United States of America; 4 Center for Computational and Integrative Biology, Massachusetts General Hospital, Charlestown, Massachusetts, United States of America; University of Utah School of Medicine, United States of America

## Abstract

Zinc Finger Nucleases (ZFNs) made by Context-Dependent Assembly (CoDA) and Transcription Activator-Like Effector Nucleases (TALENs) provide robust and user-friendly technologies for efficiently inactivating genes in zebrafish. These designer nucleases bind to and cleave DNA at particular target sites, inducing error-prone repair that can result in insertion or deletion mutations. Here, we assess the relative efficiencies of these technologies for inducing somatic DNA mutations in mosaic zebrafish. We find that TALENs exhibited a higher success rate for obtaining active nucleases capable of inducing mutations than compared with CoDA ZFNs. For example, all six TALENs tested induced DNA mutations at genomic target sites while only a subset of CoDA ZFNs exhibited detectable rates of mutagenesis. TALENs also exhibited higher mutation rates than CoDA ZFNs that had not been pre-screened using a bacterial two-hybrid assay, with DNA mutation rates ranging from 20%–76.8% compared to 1.1%–3.3%. Furthermore, the broader targeting range of TALENs enabled us to induce mutations at the methionine translation start site, sequences that were not targetable using the CoDA ZFN platform. TALENs exhibited similar toxicity to CoDA ZFNs, with >50% of injected animals surviving to 3 days of life. Taken together, our results suggest that TALEN technology provides a robust alternative to CoDA ZFNs for inducing targeted gene-inactivation in zebrafish, making it a preferred technology for creating targeted knockout mutants in zebrafish.

## Introduction

**Figure 1:**
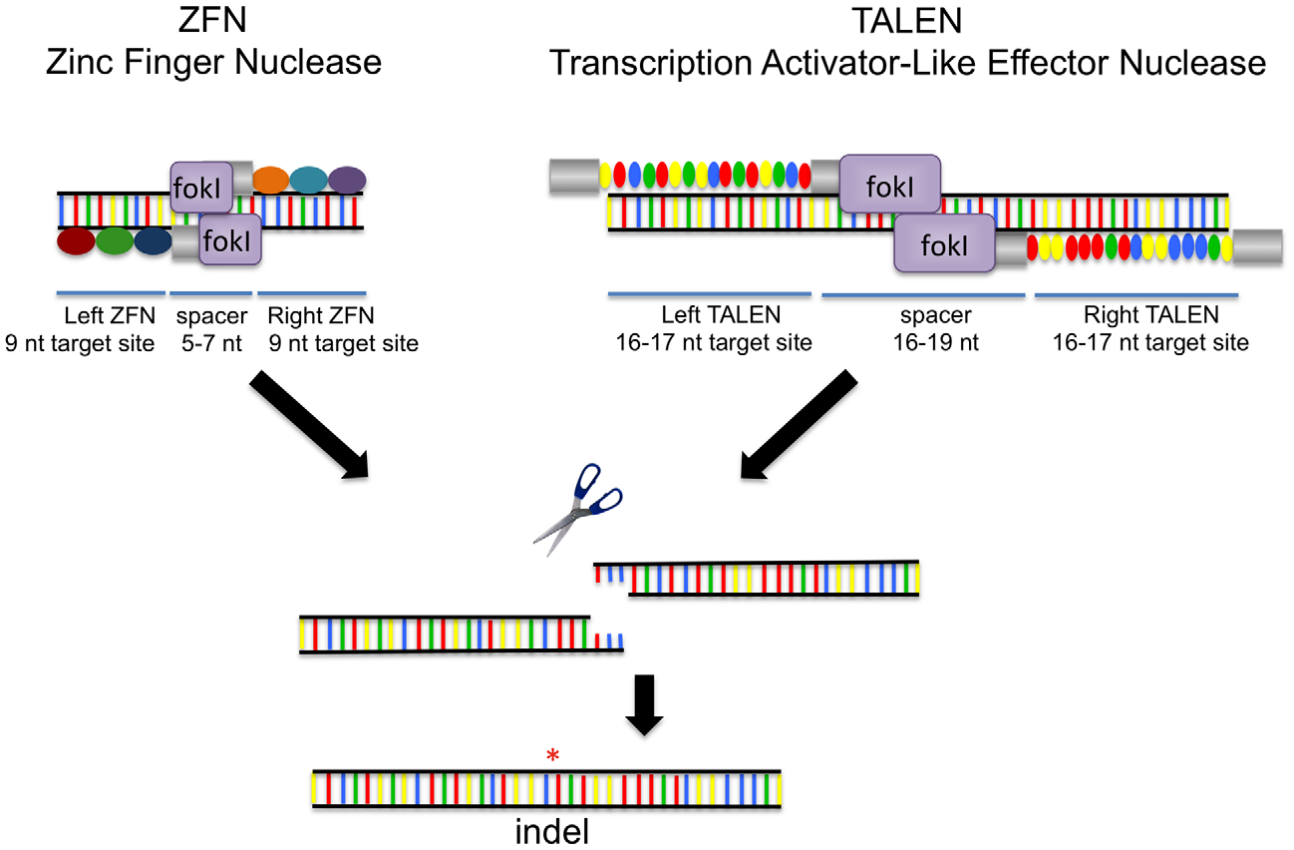
Genome engineering using ZFNs and TALENs. ZFNs utilize DNA binding domains that recognize ∼3 bp sequences and are joined together to create arrays that can target specific DNA sequences. TALENs bind DNA using TAL effector repeat domains derived from *Xanthomonas* that recognize individual nucleotides. These TALE repeats are ligated together to create binding arrays that recognize extended DNA sequences. Each ZFN or TALEN binds to a half-site with dimeric FokI nuclease domains cleaving the DNA within the intervening spacer region. The mechanism responsible for inducing DNA mutations is identical using either methodology, where nuclease-induced double stranded DNA breaks are repaired by error-prone non-homologous end joining (NHEJ) resulting in the creation of insertion or deletion mutations (indels).

Recent advances in genome engineering using Zinc Finger Nucleases (ZFNs) and Transcription Activator-Like Effector Nucleases (TALENs) have facilitated the creation of targeted gene knockout mutations in zebrafish [1], [2], [3], [4], [5], [6], [7], [8]. ZFNs consist of an engineered array of zinc fingers fused to the non-specific FokI nuclease domain and function as dimers to introduce targeted DNA double-strand breaks (DSBs). Each zinc finger binds to approximately three base pairs (bps) of DNA and a ZFN monomer commonly utilizes three to six zinc finger motifs to bind 9–18 bp target DNA. By contrast, TALENs bind to DNA through a highly conserved 33–35 amino acid transcription activator-like (TAL) effector repeat domain found in the plant pathogen *Xanthomonas*. Each TAL effector repeat domain binds to a single bp of DNA with specificity associated with the identity of two amino acids within the repeat known as repeat variable di-residues (RVDs) [9], [10], [11]. TAL effector repeats can be joined together into extended arrays that can bind to longer DNA sequences. As with zinc fingers, TAL effector repeats can be fused to the FokI nuclease domain to create TALENs capable of cleaving DNA as a dimer. DSBs induced by either ZFNs or TALENs can be repaired by non-homologous end joining (NHEJ) – an error-prone process that results in the creation of insertion or deletion mutations (indels) that can shift the translational reading frame and frequently lead to premature termination (Figure 1).

ZFNs have been successfully used to create targeted DNA mutations within somatic cells of zebrafish and have led to the production of heritable loss-of-function mutations [4], [5], [6], [12], [13]. For example, Foley et al. used Oligomerized Pool ENgineering (OPEN) to create ZFN pairs that induce targeted insertions and deletions into five endogenous zebrafish genes with high efficiency [4]. The process of creating ZFNs by OPEN requires selection-based methods to identify zinc finger arrays that bind to target DNA sequences with high efficiency [14], [15] - a process that is labor intensive and thus far not adapted for large-scale production. A simple alternative design-based method for assembling ZFNs known as Context Dependent Assembly (CoDA) was recently described. CoDA does not require selection and is therefore simpler and easier to perform for most researchers. ZFNs made by CoDA were successfully used to modify 12 endogenous zebrafish genes [2]. However, the success rate and mutagenic activities of CoDA ZFNs are generally lower than that of OPEN ZFNs. Moreover, using currently available reagents, sites for which CoDA ZFNs can be made occur only once in every ∼500 bps of random DNA sequence, thereby limiting the ability of this platform to precisely target mutations within a given gene [2].

TALENs have recently been shown to provide an alternative to ZFNs for introducing DNA mutations into zebrafish. For example, Huang et al. used TALENs to successfully target two genes for heritable gene inactivation [8]. Sander et al. also constructed four TALENs designed to target DNA sites in two endogenous genes [1]. This report showed that TALENs were capable of mutagenizing the same genomic regions as OPEN ZFNs with similar overall efficiency [1]. However, OPEN ZFNs targeting these regions also exhibited exceedingly high rates of mutation, ranging from 26–29% in somatic zebrafish cells. To date, it is unknown how efficiently TALENs induce DNA mutation at target genes for which corresponding ZFN mutation rates are below 5%. Moreover, each of these published reports focused only on TALENs that could successfully induce somatic DNA mutations when microinjected as RNA into one-cell stage zebrafish and did not discuss if additional TALENs were screened that failed to induce mutation, making it difficult to ascertain the overall success rate of TALENs for inducing mutagenesis in zebrafish. Finally, it has been suggested that TALENs exhibit an expanded targeting range when compared to CoDA ZFNs; however, TALENs have not been designed to target the methionine translation start site of endogenous zebrafish genes nor has the technology been assessed for inducing DNA mutations at sites that could not be targeted by ZFNs.

Here, we compare the abilities of TALENs and CoDA ZFNs to induce somatic mutations in zebrafish. TALENs possess higher targeting ranges compared with CoDA ZFNs and therefore could be designed to target specific regions of the genome including the methionine translation start site in multiple target genes. We also report higher success rates for TALENs at inducing mutations at any given DNA target site when compared with CoDA ZFNs. In addition, the efficiency of NHEJ-mediated mutagenesis within the same target gene is significantly higher using TALENs compared with CoDA ZFNs. We conclude that TALENs provide a superior platform to CoDA ZFNs for inducing targeted DNA mutations in zebrafish and that this platform will therefore play a major role in engineering the next generation of knockout fish designed to uncover important pathways in development, disease, and cancer.

## Materials and Methods

### Ethics Statement

This study was approved by the Massachusetts General Hospital Subcommittee on Research Animal Care – OLAW Assurance # A3596-01 under protocol #2011N000127.

### Synthesis of TALEN and ZFN constructs

TALENs and ZFNs were designed using the ZiFiT Targeter software (http://zifit.partners.org/) [16]. DNA fragments encoding ZF arrays were synthesized by GenScript, cloned by BamHI/XbaI digest into FokI expression vectors as previously reported [17]. The FokI expression vectors encode previously described FokI heterodimer mutants [18], either FokI^Q486E/I499L;E490K/I538K^ (EL/KK) or FokI^Q486E/I499L/N496D;E490K/I538K/H537R^ (ELD/KKR). DNA fragments encoding engineered TALE repeat arrays were constructed using the FLASH assembly method [19]. All TALENs were built on a previously described framework [2], [20] that has now been used to efficiently modify various endogenous genes in *C.elegans* [21], rats [22], zebrafish [1], and human somatic [19], [20] and pluripotent stem cells [23]. Each TAL effector repeat array was designed to target 16 or 17 bp DNA half-sites. Fragments encoding TAL effector repeat arrays were cloned into wild-type FokI expression vectors (available from Addgene: http://www.addgene.org/talengineering/expressionvectors/) as previously described [1]. The C-terminal 0.5 TALE repeat domain varies in these different expression vectors. All TALEN expression vectors were verified by sequencing (Eurofins Operon).

### RNA transcription and injection

RNA was synthesized using the Ambion mMACHINE T7 kit as described [17]. Briefly, ZFN or TALEN DNA was linearized with PmeI (NEB), purified by Qiagen QiaQuick kit. 500 ng of purified linearized ZFN or TALEN DNA was transcribed according to the manufacturer's instructions. RNA encoding each TALEN arm were combined and resuspended in nuclease free water at a concentration of 250 ng/µl. One pl (125 pg RNA of each of the two ZFN or TALEN arms, 250 pg total RNA) was injected into the single cell zebrafish embryo. For some injections 4× concentration of RNA was used (1 ng total RNA).

### Calculating mutation rates in somatic cells of microinjected fish

Mutation rates were determined as described (Figure S1) [17]. Microinjected zebrafish embryos were raised to 3 dpf. Genomic DNA was extracted from 12 larvae and treated with SDS lysis buffer (10 mM Tris, 10 mM EDTA, 200 mM NaCl, 0.5% SDS, 100 mg/ml proteinase K) for 2 h at 50°C. Genomic DNA was purified using phenol-chloroform extraction and PCR was performed using primers that span the target site of interest (Table S1). Genomic PCR products were purified by Qiagen MinElute kit, cloned into TOPO vector (Invitrogen TOPO TA kit), and transformed into Mach1 bacterial cells. Plasmid purification and sequencing by T3 primer were performed by the MGH DNA core facility. DNA sequence alignments were performed using LaserGene DNAStar SeqMan program and DNA mutation rate calculated as the number of mutant sequences divided by the total number of sequences that covered the target region multiplied by 100. Single insertions, deletions, or substitutions were not considered mutations in this analysis as these could result from PCR or sequencing artifact. Sequences that failed to include both sides of the targeting region were excluded from our analysis.

### Zebrafish husbandry and toxicity estimates

Zebrafish were raised according to standard procedures [24]. Specifically, microinjected zebrafish embryos and uninjected control fish from the same clutch were analyzed for dead and deformed embryos at 24 hpf and 3 dpf [17], [25]. Tu/AB mixed strain was used for all injections, however, these fish exhibited a single-nucleotide polymorphism (SNP) within the *jak3* targeting site. Thus, *jak3* ZFNs or TALENs were also microinjected into AB-strain fish that lacked this SNP.

### Statistics

DNA mutation rates are shown as mean percentages +/− standard error. For comparing CoDA ZFNs to our TALEN results, the highest reported mutation rates for each ZFN pair were used, providing a conservative analysis of differences between mutation rates between CoDA ZFNs with TALENs. Fisher's Exact test was used in comparing the numbers of clones that contained wild-type or mutant sequences between TALEN and ZFN pairs for a given gene of interest.

A Fisher's exact test was also performed to assess the difference in number of successes between CoDA ZFNs and TALENs. To account for the possibility of false negatives in our analysis, we established a detection limit of 3.5%. At this threshold, ≥84 sequences would be required to identify a >3.5% mutation rate with >95% confidence. Based on these criteria, 5 data points were eliminated from subsequent analysis. The remaining 44 data points, 38 ZFNs and 6 TALENs, were binned into two categories, “Positives” (indel mutations detected) and “Negatives” (indel mutations not detected and rates can be confidently predicted to be below 3.5%), to generate a 2×2 contingency table. The null hypothesis that the distribution of data points within this table is random was assessed using the Fisher's exact test.

## Results

### The use of modified heterodimeric FokI nuclease domains does not enhance the mutagenic activities of CoDA ZFNs

In a previously published study [2], zebrafish genes were modified by CoDA ZFNs that harbored heterodimeric FokI variants bearing mutations at positions Q486E/I499L and E490K/I538K (hereafter referred to as EL/KK) [18]. However, more recent work has suggested that the activities of ZFNs can be enhanced by using another pair of heterodimeric FokI variants bearing mutations at Q486E/I499L/N496D;E490K/I538K/H537R (hereafter referred to as ELD/KKR) [26]. These ELD/KKR variants were reported to increase target DNA cleavage rates *in vitro* and in human cells due to the introduction of additional mutations that strengthen the dimerization of the FokI subunits [26].

To directly address if the ELD/KKR heterodimers could result in higher mutation rates in zebrafish, we sought to test these variants using ZFNs made by CoDA. To perform this comparison, we chose nine CoDA ZFNs that had been previously tested using the EL/KK FokI variant. Six of these nine had previously exhibited detectable mutagenesis at their intended endogenous gene target (range of ∼0.9 to 16.6%) and three had failed to show detectable mutagenesis activities as assessed by low-throughput Sanger sequencing approaches (Figure S2 and Table S2) [2]. Use of the ELD/KKR FokI domains did not enhance the activities of the CoDA ZFNs we tested. ZFNs that had higher mutagenic activity levels as EL/KK ZFNs still showed comparable levels as ELD/KKR ZFNs (*rag2b* and *actinin* in Figure S2 and Table S2). Interestingly, our re-testing of these nine CoDA ZFNs bearing the EL/KK variant using the same low-throughput Sanger sequencing method as the original report did not consistently detect the mutagenic activities of ZFNs. One ZFN pair, *tp53*, induced mutations with the ELD/KKR FokI but not EL/KK FokI. However, the level of this *tp53*-targeted ZFN was low (2.5%), consistent with the lack of a significant difference in activity relative to the EL/KK ZFNs (Figure S2 and Table S2). We conclude that use of the modified ELD/KKR FokI variants does not appear to substantially improve mutation rates of CoDA ZFNs in somatic cells of zebrafish.

### CoDA ZFNs that induce DNA target site mutations can be identified without pre-screening by the bacterial two-hybrid assay

In a previous report, zinc finger arrays made by CoDA were first pre-screened using a bacterial two-hybrid (B2H) reporter assay before being used as ZFNs [2]. Finger arrays that activated transcription by ≥ three-fold in the B2H reporter assay were shown to have a ∼50% success rate at inducing DNA mutations when incorporated into ZFN pairs and assessed for mutagenic capability in mosaic zebrafish. Assuming that 75% of monomeric CoDA zinc finger arrays activate ≥ three-fold in the B2H system, one would expect that ∼56% ( = 75%×75%) of pairs would meet this criterion. Of these pairs, one would expect that ∼28% ( = 56%×50%) of the resulting CoDA ZFN pairs would show detectable mutagenesis activity. Given these theoretical success rates, it was suggested that pre-screening with the B2H might not be necessary if one was willing to accept a lower success rate in identifying active ZFN pairs that cleave with efficiencies of ≥1% [2]. To test this possibility, we designed 17 CoDA ZFN pairs using the ZiFiT Targeter software and cloned DNA encoding these zinc finger arrays into vectors that contained the ELD/KKR FokI variants. Three of 17 (∼18%) non-selected CoDA ZFN pairs induced indels at the target site within the population of the clones we sequenced, with rates ranging from 1.1–3.3% (Figure S2 and Table S3). We conclude that, as previously predicted, ZFNs designed by CoDA that are not pre-screened using the B2H reporter assay can efficiently induce DNA mutations in mosaic animals.

### Comparisons of CoDA ZFNs and TALENs for targeting endogenous zebrafish genes

**Figure 2:**
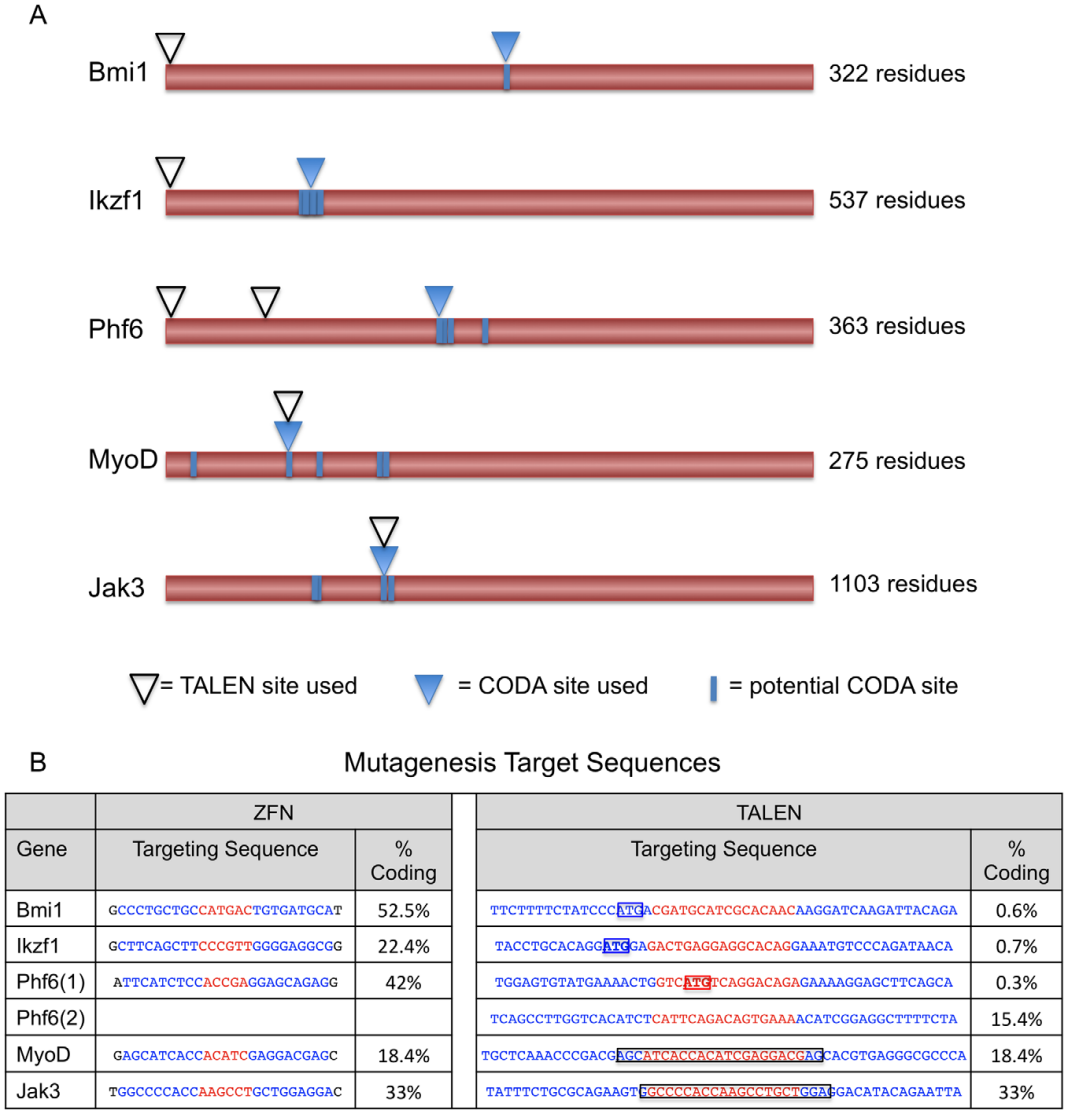
CoDA ZFN and TALEN targeting sites used for assessing mutation rates across platforms. A) Schematic comparing target sites for ZFNs generated using CoDA compared with TALENs. Peptide sequence is represented by brown bar. Arrowheads denote sites used to target ZFNs (blue) compared to TALENs (open white). All potential CoDA sites are shown as blue hashes within the coding sequence. B) Target sequences for ZFNs and TALENs. Blue text indicates DNA binding site of ZFN or TALEN, red text indicates spacer region. The *jak3* and *myoD* targeting sites are overlapping for both ZFNs and TALENs, shown by boxed text within the TALEN sequence. Start codons are shown in boxed text for TALENs designed to *bmi1, ikzf1*, and *phf6*. Target sites are denoted by their percentage distance within the coding region (% coding = % peptide sequence).

We initially compared the targeting ranges of CoDA ZFNs and TALENs for inducing mutations in five endogenous zebrafish genes: *B lymphoma Mo-MLV insertion region 1 homolog Polycomb RING finger protein (bmi1), Ikaros Family Zinc Finger* (*ikzf1*), *Plant Homeodomain Finger 6* (*phf6*), *Myoblast determination protein 1 (myoD),* and *Janus Kinase 3 (jak3)*. Using the publicly available ZiFiT Targeter software program, we identified an average of 3.8 potential CoDA ZFN sites per gene (Figure 2), an average target site frequency of 1 in 387 bp within the coding sequence is consistent with the previously reported CoDA targeting range of one site in every 400 bps in the zebrafish exome [2]. Of these various targets, CoDA ZFN sites were chosen to avoid pyrimidine-rich sites and to target as early in the 5′ open reading frame as possible. CoDA ZFNs were designed to DNA target sites ranging from 18% to 57% into the amino acid coding sequence for the genes analyzed (Figure 2). By contrast, TALENs could be designed to the methionine translation start site for all five genes using targeting parameters developed by the Joung lab (Figure 2 and data not shown) [19].

**Figure 3:**
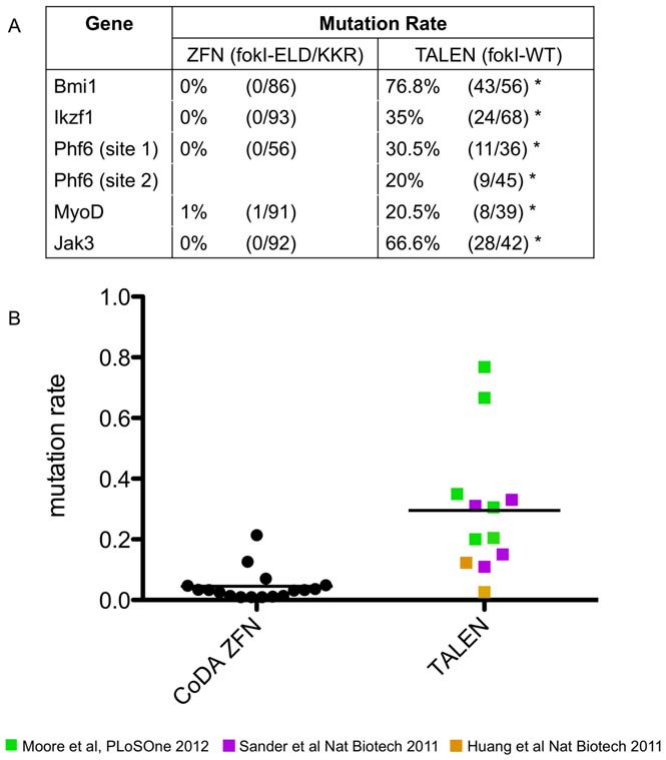
TALENs exhibit high mutation rates. A) Table showing somatic mutation rates of ZFNs and TALENs for five genes. Mutation rate was calculated as number of mutant sequences divided by the total number of sequences analyzed for a given target region. Raw sequence scores are shown in parentheses. Each TALEN had a significantly different mutation rate compared to ZFNs as assessed by Fisher Exact Test (p<0.001 denoted by asterisks). B) Plot of somatic mutation rates of CoDA ZFNs and TALENs in zebrafish [1], [2], [8]. The average mutation rate of all TALENs was higher than that of CoDA ZFNs. ZFNs with mutation rates of 0% were excluded from this analysis. Datum points of TALENs from different research groups are distinguished by color.

**Figure 4:**
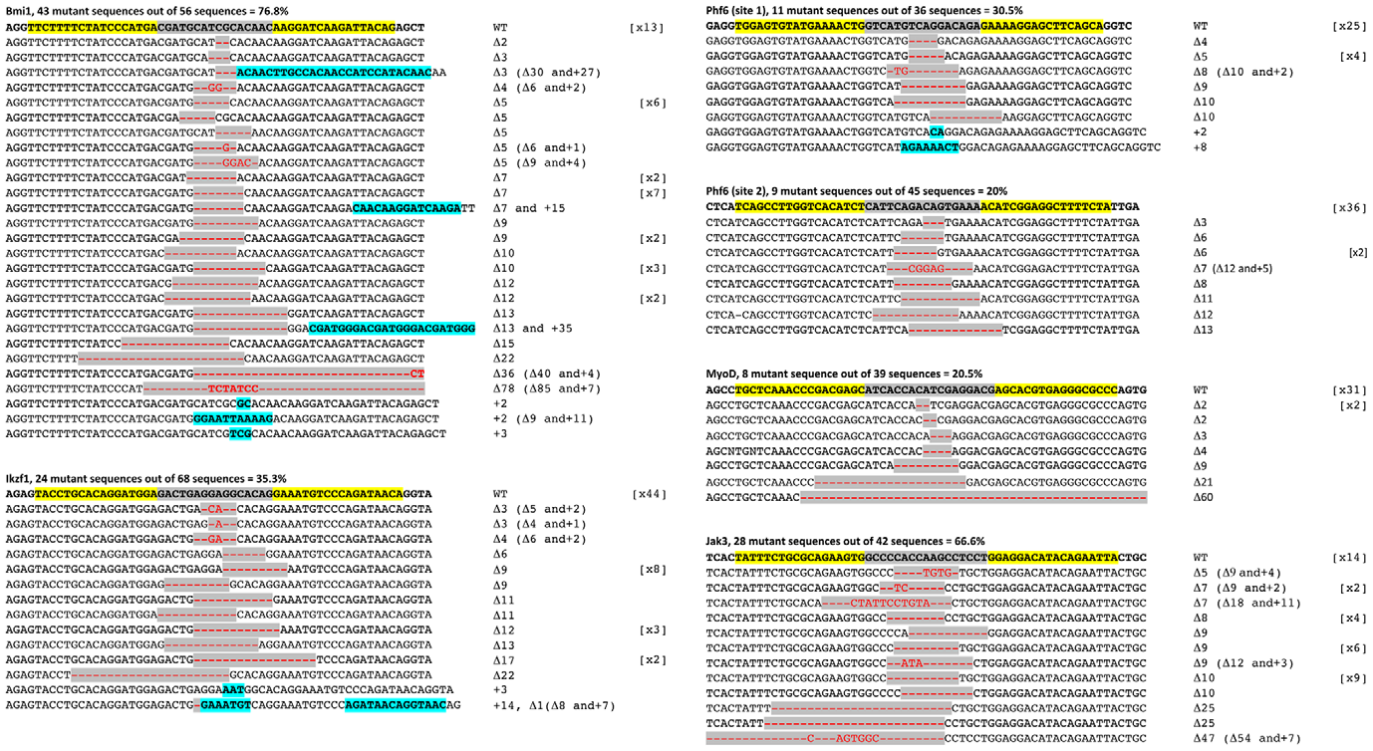
Sequences of somatic zebrafish gene mutations induced by TALENs. Mutant sequences were aligned to the wild-type sequence. The length and frequency of indels are noted to the right. The target site is shown at top with each TALEN half-site highlighted in yellow and the spacer sequence highlighted in gray. Deletions (Δ) are shown in red with gray highlight, insertions (+) are shown in blue.

To compare success rates of CoDA ZFNs and TALENs for mutagenizing target sites, we assessed the activities of nucleases targeted to both the same and different regions of endogenous zebrafish genes. For the *bmi1, ikzf1*, and *phf6* genes, we designed TALENs targeting the translation start site (Figure 2). An additional TALEN pair for *phf6* was designed to target a site in the second exon of this gene. For *jak3* and *myoD*, we designed TALENs to target similar genomic sequence as CoDA ZFNs (Figure 2) to ensure that differences in ZFN and TALEN mutation rates did not result from altered chromatin structure or DNA methylation status at the target site of interest. Of the five CoDA ZFNs tested, only the ZFN targeted to the *myoD* locus showed evidence of somatic mutation at 3 dpf (Figure 3 and Figure S2). The CoDA ZFN pair designed to the *jak3* gene showed mutagenic activity in a previously published report [2] but was not detected in our current analysis. This failure to detect mutagenic activity for the *jak3* CoDA ZFNs is likely due to the sampling limitation of our Sanger sequencing assay (Table S2 and Discussion). By contrast, all TALENs designed to these same genes led to highly efficient somatic mutation within embryos, ranging from 20%–77% (Figure 3 and Figure 4).

To more broadly compare success rates of CoDA ZFNs and TALENs for mutagenesis of a given target in zebrafish, we performed an analysis of results of CoDA ZFNs obtained among data presented here and within multiple research groups in the community, including ZFNs that were and were not pre-screened by B2H-assay, and compared to our data for TALENs [1], [2], [8]. We find that TALENs are significantly more likely to induce detectable DNA mutations than CoDA ZFNs with 17 of 38 ZFNs inducing DNA mutations at the target site of interest compared to 6 of 6 TALENs reported here (p = 0.02, Fisher's Exact test, Table S4 and Materials and Methods). Our results suggest that the success rate for inducing mutations at a given target site is higher for TALENs than for CoDA ZFNs.

TALENs also induce higher rates of mutagenesis at their endogenous gene targets than CoDA ZFNs. All TALEN pairs exhibited significantly higher mutation rates when compared with CoDA ZFNs to the same five genes, albeit at different DNA target sites (Fisher's Exact Test, p<0.001 for individual comparisons between genes, Figure 3A). When compared to published results, there is also a difference between CoDA ZFNs and TALENs with CoDA ZFN mutation rates being 4.5+/−1.3% (n = 17) compared to the TALEN mutation efficiency of 29.5+/−6.4% (n = 12, Figure 3B and Table S4) [1], [2], [8]. Thus, on average, TALENs are more than six-fold more efficient at inducing targeted DNA mutations in developing zebrafish.

### TALENs can induce large mutations that produce early frame shift mutations and/or protein termination

**Figure 5:**
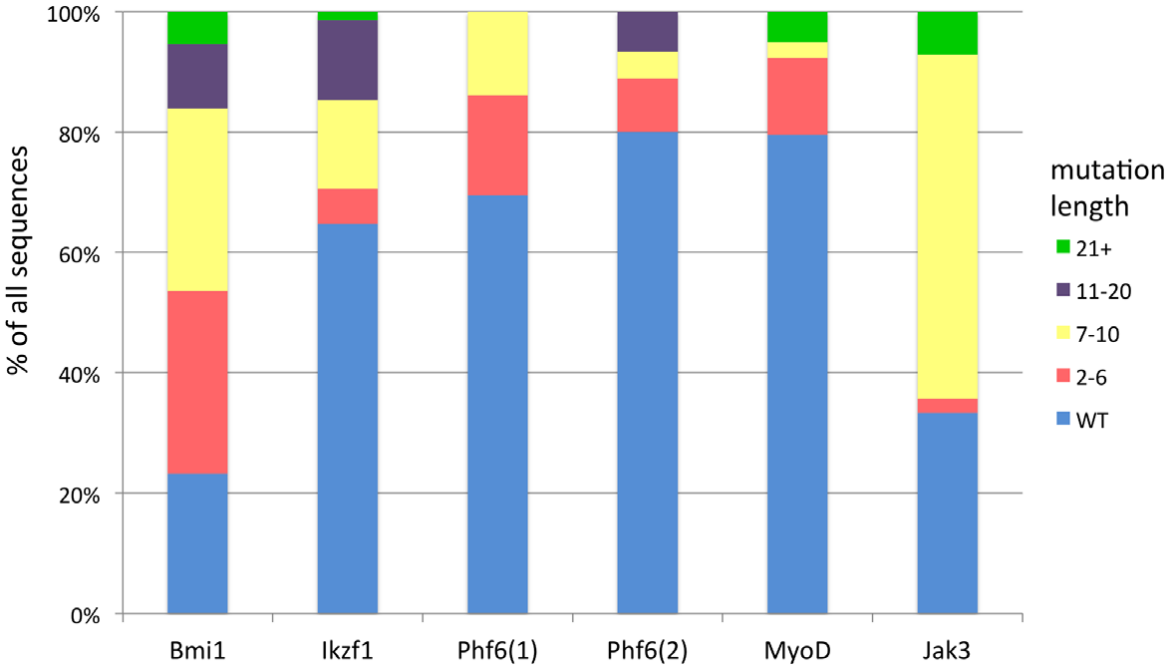
TALENs cause a wide diversity of DNA mutations. Indels were classified according to length. The frequency of different length mutations is shown as a percentage of all sequences.

**Figure 6:**
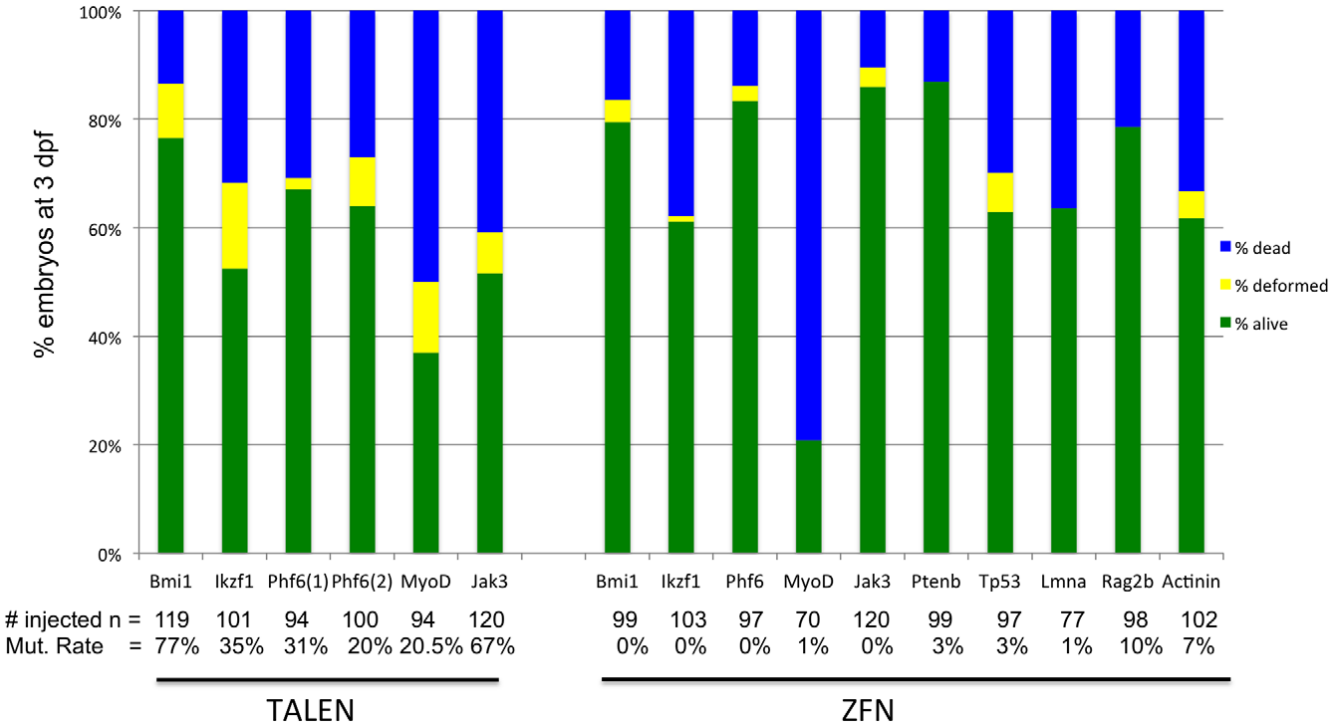
TALENs and ZFNs exhibit similar toxicity profiles. The cumulative percentage of embryos that were dead or deformed by 3 days post-fertilization is denoted. CoDA ZFNs from the same genes are shown on the right. The number of fish examined for toxicity is shown below; the mutation rate is derived from a pool of 12 embryos.

TALENs induce mutations with a diverse range of lengths (Figure 4 and 5). TALEN mutant sequences included indels ranging from two to 78 bps (Figure 5). We find that TALENs induce 11.5+/−1.4 bp indels on average (n = 76 independent mutations from all six TALENs). More than half of all mutant sequences were unique across TALEN targets (data not shown), suggesting that TALENs are able to cleave genomic DNA during late stages of development and in multiple cell types within animals. In our dataset, induced mutations would presumably create LOF alleles 66% of the time, eliminating the ATG start site or inducing early frameshift followed by stop codons within 50 amino acids of the DNA target site. Finally, high mutation rates would not be useful if the cost were high toxicity. Overall, TALENs had largely similar toxicity profiles when compared with CoDA ZFNs with more than half of all embryos injected with TALEN RNA surviving to 3 days post-fertilization (Figure 6).

## Discussion

One major advantage of TALENs is their higher targeting range relative to CoDA ZFNs. For example, the DNA targeting range for CoDA ZFNs is limited by the availability of pre-selected zinc finger units that can bind to each of the possible 3 bp sequences. CoDA ZFNs have been reported to target 1 in 400 bp of sequence in the zebrafish exome [2]. By contrast, TALENs utilize a simpler “code” of binding where each TALE repeat recognizes one nucleotide. Recent work has shown that TALENs exhibit a conservative targeting range of three TALEN pairs for every bp of random DNA sequence [19] and can therefore in nearly all cases be easily designed to essentially any region of a gene including the translational start site. Building on these recent findings, we utilized TALENs to specifically ablate the methionine start site in three endogenous zebrafish genes and induced DNA mutations within the coding sequence of three genes. Such flexibility in target design will likely facilitate the creation of knockout zebrafish and the production of allelic deletion mutations to assess complex structure-function relationships in live animals.

Our results also suggest that TALENs possess a higher success rate for targeting a given gene for mutation than CoDA ZFNs. For example, all six TALENs tested in our study exhibited high rates of DNA mutagenesis at the target site of interest while fewer than half of all CoDA ZFNs induced indels within the population of sequenced clones. These data also suggest that TALENs are more likely to successfully target a given DNA target for mutagenesis than CoDA ZFNs, irrespective of whether the ZFN pairs were pre-screened by B2H assay.

The average efficiency with which TALENs induce somatic mutations is also higher than that of CoDA ZFNs. In combining data from our work and those previously reported by Sander et al., 2011, we find that CoDA ZFNs exhibit an average mutation rate of 4.5+/−1.2% at the target DNA sequence (n = 17, data includes both pre-selected and non-selected ZFNs that exhibited detectable mutation rates as assessed by Sanger sequencing, Table S4). By contrast, an average somatic mutation frequency of 29.5+/−6.4% could be achieved using TALENs [1], [8]. Most importantly, all TALEN pairs tested in our study exhibited significantly higher mutation rates than compared with CoDA ZFNs designed to the same five genes, albeit at different DNA target sites. We note that the difference we observe may be partly attributable to the use of the wild-type homodimeric FokI cleavage domain within the TALENs while CoDA ZFNs used one of two different heterodimeric FokI domains [18], [26]. Although the TALENs used here may have higher off-target mutation events due to the use of homodimeric FokI, it is likely that founders can be outcrossed to reduce the mutation load at other alleles. Such approaches are currently used to eliminate off-target ENU induced mutations in zebrafish isolated from genetic screens and TILLING. Future studies will be required to assess rates of off-target TALEN mutations in zebrafish and the ability to curb these effects using heterodimeric FokI cleavage domains.

We note that the high success rates and mutagenesis frequencies we observed in this report were obtained using TALENs built on a specific architecture of TAL effector repeats and N- and C-terminal extensions of TAL effector sequence beyond the repeats. This particular architecture was also used in a previous study in which TALENs induced high rates of mutagenesis in zebrafish [1] and in other studies where endogenous genes from *C.elegans* [21], rats [22], human somatic [19], [20] and pluripotent stem cells [23] were efficiently modified with TALENs. Interestingly, another earlier report that used TALENs to modify zebrafish genes [8] found lower frequencies of mutagenesis using a different amino acid framework for the TAL effector repeats (Figure 3, Table S4). A variety of different architectures for making TALENs have been described in the literature [27], [28], [29], [30], [31], [32] and an important question for future investigation will be to determine if TALENs built using these other approaches will also exhibit the same high success rates and mutation frequencies that we observed in this report.

Our findings also suggest limitations to low-throughput Sanger sequencing assays to assess targeted nuclease activities. The small numbers of sequences sampled with this assay limit the reliable detection of nucleases that induce low-frequency DNA mutations. For example, using this assay, we did not detect somatic DNA mutations in animals injected with CoDA ZFNs to three different genes (*jak3*, *bmpr2a*, and *grip1*) that had previously been reported to induce mutations at rates ranging from 0.9% to 3.3%. Conversely, we were able to detect mutations induced by CoDA ZFNs targeted to *tp53* that had tested negative in previous experiments. These results demonstrate that assessing mutation rate by Sanger sequencing is prone to sampling artifact and that this method may overestimate failure rates. This finding is not entirely surprising given that sequencing of 84 samples can only detect mutation frequencies of >3.5% with 95% confidence and sequencing of 300 samples would be required to reliably detect mutation frequencies of 1%. High-throughput next-generation sequencing may therefore provide a more sensitive assay for assessing true mutation rates where somatic DNA mutation rates have been reported as low as 0.01% for a subset of ZFNs [6]. Moreover, nucleases that induce low somatic mutation rates are likely to still be of value to investigators because heritable germ line mutations have been observed with ZFN pairs that have somatic mutation rates as low as 0.1% as assessed by next generation sequencing [6]. Despite the limitations of Sanger sequencing in detecting low level mutation rates for CoDA ZFNs and the likely underestimation of the number of ZFNs that can induce mutations in somatic cells of zebrafish, it is clear that such approaches identified significantly high mutation frequencies at genomic DNA target sites modified by TALENs. Taken together, these data strongly argue that TALENs induce mutations more efficiently than CoDA ZFNs and will likely require fewer animals to be screened to identify founder fish containing mutations of interest.

Looking to the future, it is likely that TALEN technology will revolutionize reverse genetics in zebrafish and will be used to create a much-needed library of loss-of-function alleles for the community. In the experiments outlined in this report, we were able to construct TALEN expression vectors within two weeks and establish somatic mutation rates for each pair within another week. Further, because TALENs cleave endogenous target DNA with high efficiencies, it is possible that TALENs may play a critical role in the development of strategies for performing efficient homologous recombination in zebrafish. Such approaches would allow precise mutations to be targeted to a gene of interest while Cre/lox technologies could provide temporal and spatial control of gene inactivation during development. In summary, our results provide support for the use of TALENs as a robust platform for engineering DNA mutations into zebrafish and suggest that TALEN technology will fast become the preferred technology for creating knockout mutants in zebrafish.

## Supporting Information

Figure S1 Schematic illustrating methodology used to assess somatic mutation rates of TALENs. DNA fragments encoding TAL effector repeat arrays were cloned into TALEN expression vectors. Each construct contained a single TALEN and was transcribed into RNA. Pairs of TALEN-encoding RNAs were microinjected into the single cell stage zebrafish embryos (250 pg total RNA injected into each embryo). TALENs induce double strand breaks (DSB) at the DNA target site. Non-homologous end joining repairs the DSB, often resulting in insertion or deletion mutations (indels) at the target site. Genomic DNA was extracted from 12 microinjected zebrafish at 3 dpf and genomic DNA fragments spanning the target site were amplified using PCR. Fragments were resolved on a gel, purified, and cloned into a TOPO vector. Clones were sequenced to assess mutation frequencies within the target region of interest. Mutation rates were defined as the number of mutant sequences divided by the number of sequences analyzed multiplied by 100.
(TIF)

Figure S2 Sequences of somatic zebrafish gene mutations induced by CoDA ZFNs. Mutant sequences were aligned to the wild-type sequence. The length and frequency of indels are noted on the right side. The target site is shown at top with each CoDA ZFN half-site highlighted in yellow and the spacer sequence highlighted in gray. Deletions (Δ) are shown in red with gray highlight, insertions (+) are shown in blue.
(TIF)

Table S1 PCR primers used to amplify genomic DNA in target regions to determine mutation rate.
(XLSX)

Table S2 The use of a modified FokI heterodimer does not alter ZFN mutation rates in zebrafish. Heterodimeric FokI^Q486E/I499L;E490K/I538K^ (EL/KK) cleavage domains [18] were compared to the modified heterodimeric FokI^Q486E/I499L/N496D;E490K/I538K/H537R^ (ELD/KKR) cleavage domains [26]. Previous work used the EL/KK FokI domains [2].
(TIF)

Table S3 Mutation rates for ZFNs designed by CoDA. Raw sequence scores are shown in parentheses. For some ZFNs, more than one RNA dose was injected, 1× = 250 pg total RNA, 4× = 1 ng total RNA.
(TIF)

Table S4 Compilation of mutation rates for ZFNs and TALENs. In the case of multiple injections, the highest rate of mutagenesis is shown for a given ZFN. Data is plotted in Figure 3B [1], [2], [8].
(XLS)
