# Supplementary figures and images for: Improved Somatic Mutagenesis in Zebrafish Using Transcription Activator-Like Effector Nucleases (TALENs)
Source: PLoS One. 2012 May 24;7(5):e37877. doi: 10.1371/journal.pone.0037877 (PMC3360007; doi:10.1371/journal.pone.0037877)

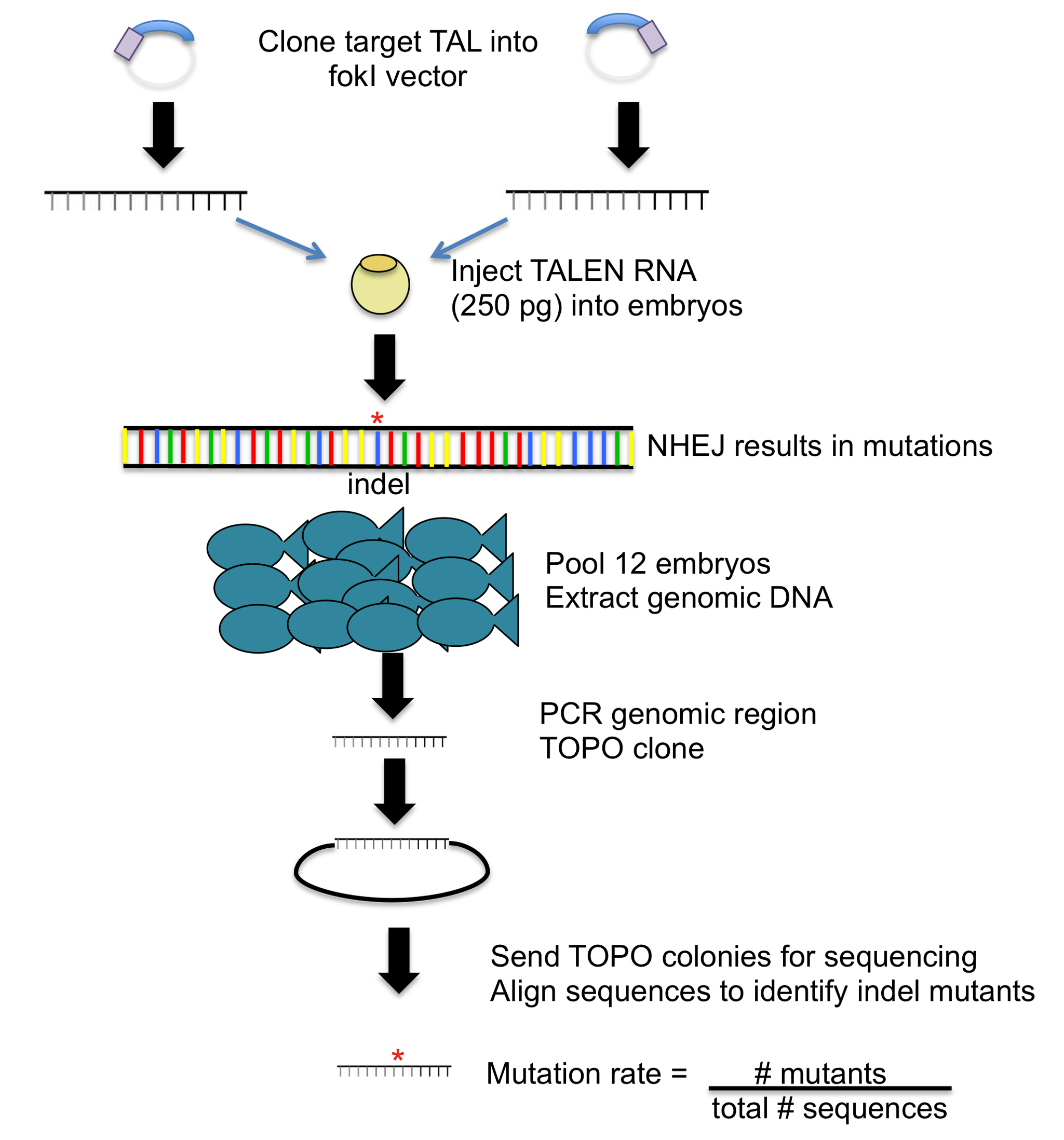

Supplement: Figure S1 — Schematic illustrating methodology used to assess somatic mutation rates of TALENs. DNA fragments encoding TAL effector repeat arrays were cloned into TALEN expression vectors. Each construct contained a single TALEN and was transcribed into RNA. Pairs of TALEN-encoding RNAs were microinjected into the single cell stage zebrafish embryos (250 pg total RNA injected into each embryo). TALENs induce double strand breaks (DSB) at the DNA target site. Non-homologous end joining repairs the DSB, often resulting in insertion or deletion mutations (indels) at the target site. Genomic DNA was extracted from 12 microinjected zebrafish at 3 dpf and genomic DNA fragments spanning the target site were amplified using PCR. Fragments were resolved on a gel, purified, and cloned into a TOPO vector. Clones were sequenced to assess mutation frequencies within the target region of interest. Mutation rates were defined as the number of mutant sequences divided by the number of sequences analyzed multiplied by 100. (TIF) [file pone.0037877.s001.tif]

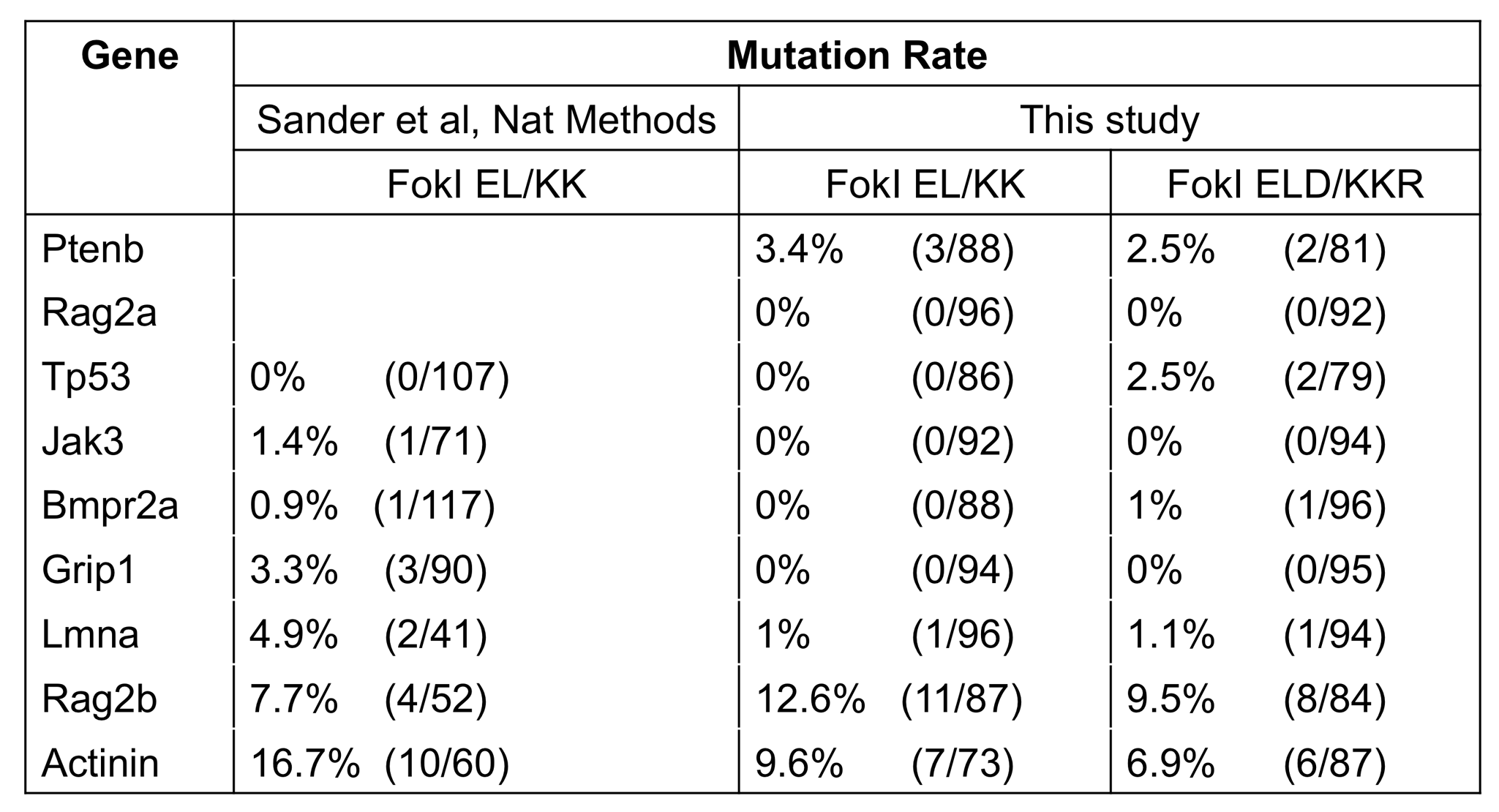

Supplement: Table S2 — The use of a modified FokI heterodimer does not alter ZFN mutation rates in zebrafish. Heterodimeric FokIQ486E/I499L;E490K/I538K (EL/KK) cleavage domains [18] were compared to the modified heterodimeric FokIQ486E/I499L/N496D;E490K/I538K/H537R (ELD/KKR) cleavage domains [26]. Previous work used the EL/KK FokI domains [2]. (TIF) [file pone.0037877.s004.tif]

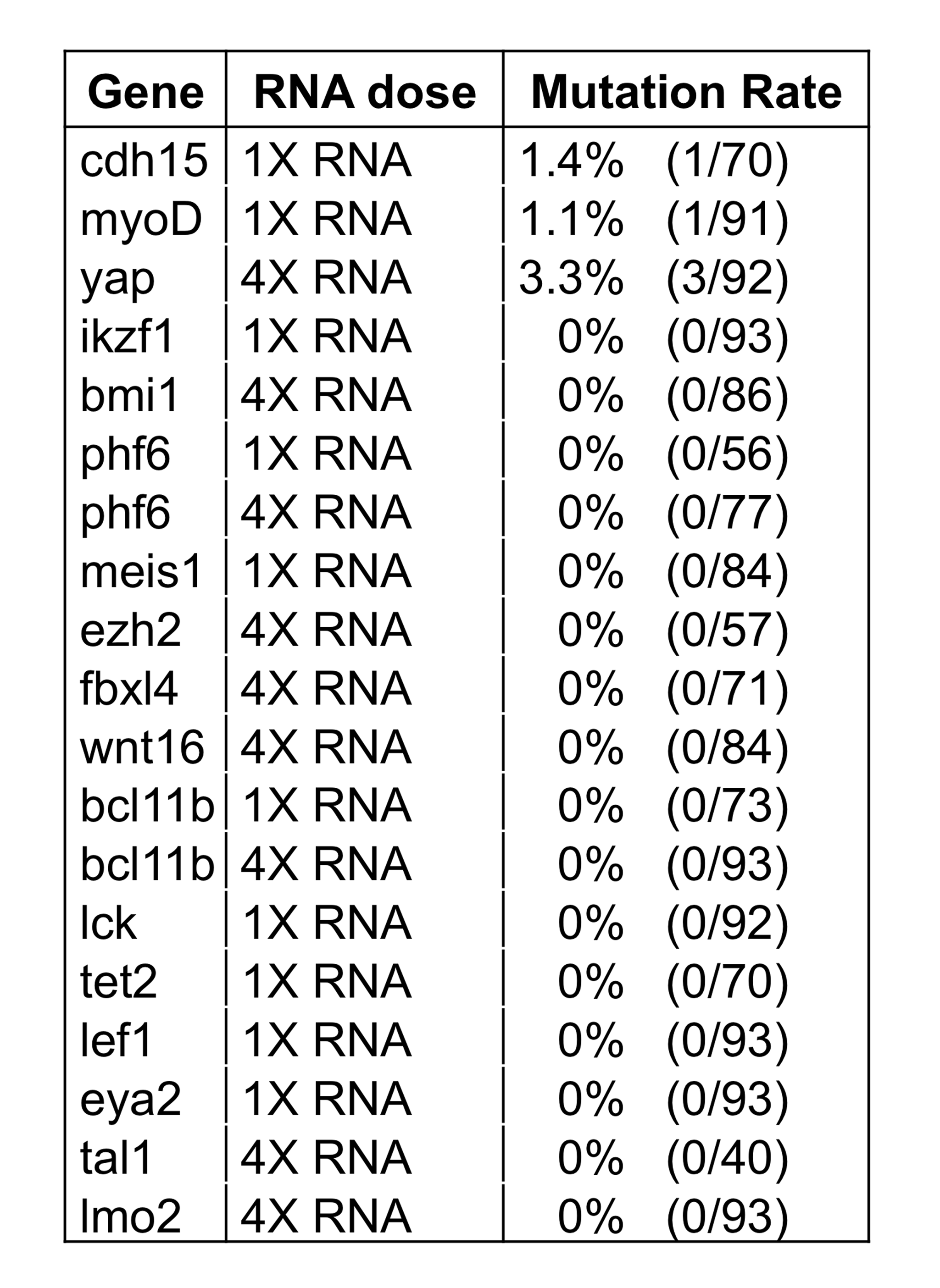

Supplement: Table S3 — Mutation rates for ZFNs designed by CoDA. Raw sequence scores are shown in parentheses. For some ZFNs, more than one RNA dose was injected, 1× = 250 pg total RNA, 4× = 1 ng total RNA. (TIF) [file pone.0037877.s005.tif]
